# The New York Institute of Stomatology

**Published:** 1897-08

**Authors:** 

**Affiliations:** The New York Institute of Stomatology


### Reports of Society Meetings.

### THE NEW YORK INSTITUTE OF STOMATOLOGY.

A REGULAR meeting of the Institute was held Tuesday evening, April 6, 1897, at the residence of Dr. J. Adams Bishop, 30 West Forty-eighth Street, New York City.

In the absence of the President, Dr. Allan, Dr. J. Morgan Howe was nominated as chairman, and acted as such.

The Secretary read the minutes of the previous meeting, which were approved.

*The Chairman.*—Gentlemen, we will first listen to Dr. A. H. Brockway, who will read a short paper on "Cleansing Teeth."

(For Dr. Brockway's paper, see page 507.)

*Dr. Brockway.*—I will say that at the request of Dr. Lord I brought over with me the instruments I use in scaling teeth, although there is nothing original about them. They are parts of several sets. I make great use of Riggs's scalers. I know that many object to them, but I would rather forego the use of any other scaler than Riggs's. Considerable practice is required, however, before one can use them satisfactorily. I had them two or three years before I had much confidence in using them. I have also parts of Dr. Allport's set, and Dr. Howe's set, and also some of Dr. Lord's, I have here, too, some of the fibre which I mentioned. If it is not familiar to all present, it ought to be, as it is one of the most useful things I know of. It is also admirable for polishing fillings.

DISCUSSION.

*Dr. F. Milton Smith.*—I would like to ask Dr. Brockway what his method is for reaching the deposits that are sometimes found three-quarters of the way down on the root. It is utterly impossible for me to remove those deposits entirely with scalers.

*Dr. Brockway.*—It is sometimes extremely difficult to reach all the deposits. I do not know that any of us can always do it, but with the instruments I have shown here I manage to do it with a tolerable degree of satisfaction. The Riggs instrument is as efficient for going deep down as any I know; but I make great use of a delicate chisel-shaped instrument, bent slightly,—one or two degrees only; with that I can feel my way down, and if there is any accretion present, I can usually discover it by the sense of touch and remove it. I have not made use of solvents, trichloracetic acid or sulphuric acid, as some do, but it may be well to do so.

*Dr. Babcock.*—I have used the dilute aromatic sulphuric acid, and have found it very beneficial. I have also used the trichloracetic acid, but did not find it as valuable. The aromatic sulphuric acid, in connection with a little tincture of capsicum, acts also as a stimulant, and where the gums are loose and inclined to bleed and be turgid, this preparation seems to accomplish a great deal, using it after the thorough mechanical removal of the deposits.

*Dr. Houghton.*—In place of pumice-stone I use Arkansas stone powder, which gives a fine finish and removes the discolorations and deposits much more effectually, in my opinion, than pumice-stone. It can be obtained from any of the jeweller supply-stores down-town. For the last few years I have had a dread of the use of the brush-wheel in cleaning teeth. Those who have a practice in which they can obtain almost any fee they ask, can use a new brush-wheel for each patient. In my estimation, there is no more dangerous source of infection than the brush-wheel. If I do use one a second time, I always soak it in electrozone. The brush comes out beautifully white, clean, and pure. I prefer above all things the little rubber cups in polishing. The other details Dr. Brockway mentioned meet with my hearty approval.

*Dr. W. St. George Elliott.*—I think most gentlemen have an erroneous impression as to what pumice-stone will do. Many people have the idea that this material is rough, and injurious because it has that feeling. Pumice-stone, while it appears to be rough, has no great amount of hardness, and one can take it on the finger and polish glass with it and it will break down into an infinitesimally fine powder and do no damage. The teeth can of course be injured by hard and injudicious brushing, but I do not think I have seen any very great injury done in that way. I use the very stiffest brush I can find, particularly in the treatment of Riggs's disease, using the brush more on the gums than on the teeth. Take any case where the gums are in a bad condition, and they can be put into a fairly healthy one by using only the brush without any medicament. Nature intended the gums to sustain the teeth and stand up under the most severe work. Civilization has given us soft food, and the gums do not do as much work as they should; they have become inflamed and do not perform their proper function.

*Dr. Z. T. Sailer.*—Does Dr. Elliott mean the friction to be on the edge of the gum or the part that covers the process? If the brush be pushed against the gingival edge resting upon the teeth absorption will follow. I can understand the philosophy of rubbing the gums hard over the process, but not over the edges of the gum, where they come in contact with the teeth.

*Dr. Elliott.*—I neglected to say that (in accordance with the custom of most present probably) when I instruct a patient to clean the teeth, it is always with a rotative movement, commencing high up on the gum, and brushing towards the cutting-edge, this after having thoroughly gone over the palatine surfaces, commencing with the lower incisors.

*Dr. Bishop.*—I agree with what Dr. Brockway says about cleaning teeth. I think we can get along without many medicaments. A good brush in a patient's hands will do more than we can. I recommend the stiffest brush that can be made. It should be made short and stiff to thoroughly clean the mouth. The teeth are the hardest material of the body, and they come up through the gum which Nature has beautifully finished around them, but which must be kept clean, and that must be done mostly by the patients, and they must have an instrument with which they can do it. Every patient I have asks, usually, "How shall I keep my teeth clean?" I tell them they have not brushed their teeth properly. My instruction is to take a dry brush before retiring, and to use it with a rotating motion to get out what food remains. No injury will be done to any mouth with a stiff brush properly used. It should be made very short, so that it will roll and reach any place in the mouth. It keeps the festoon of gum clean, and during the hours of sleep comparatively little harm can result.

*Dr. Lord.*—I do not understand how these gentlemen can say that they have never seen any harm come from the use of the brush. I have seen teeth brushed to death,—that is, the teeth became furrowed by too frequent use of stiff brushes, and not unfrequently the gums brushed away from the necks and roots of the teeth. I have sometimes thought it would be just as well if brushes had never been invented or used, as they are depended upon for cleansing the teeth in order to prevent decay, which they will not do, at least to any considerable extent. We hear persons say that they cannot understand how or why their teeth decay, for they brush them two or three times a day. They do not seem to realize or understand that there are other and more efficient means of keeping the teeth clean, particularly those surfaces which are most liable to decay. Of course, we need to use a brush say once a day, not oftener, with a little soap and an impalpable powder, that the teeth may be kept clean and polished,—more for appearance than otherwise. I prefer tooth-powder in the form of tablets, used by crushing them between the teeth and kept in the mouth until the mass becomes a paste, and then placed on the brush with the tongue, not wetting the brush. I have liked very much a preparation of chalk called velvet chalk, which comes in balls and should be used in the same way as the tablets.

It is most important that we should thoroughly teach our patients how to keep the teeth clean, for this we know is the most efficient means of preventing decay, and we should explain to them the reason why.

It is my practice to pass an instrument between all the teeth, however close or tight they may be together. This may readily be done with an instrument prepared for the purpose, and with it I use pulverized pumice-stone to polish the proximate surfaces. This allows the floss-silk to be used with ease and comfort, which is really the only means of cleansing the contact surfaces when the teeth are tight together. When there is more or less space, folded tape or an untwisted cord of suitable size should be used, so that the spaces may be kept clean and the sides of the teeth polished.

*Dr. J. F. D. Hodson.*—I wish to antagonize what Dr. Elliott has just said. I have seen very serious trouble produced upon the teeth, and upon the gums as well, that I am very sure was caused by the brush, and nothing else. I am decidedly of the opinion that both the teeth and gums are in many cases *scrubbed* too much, patients often boasting that they spend half an hour in the process each time of brushing their teeth. It is of all things what I would *not* have them do. I point out to them that the *oftener* they brush them the better they will please me, but that, after conscientiously going once or twice over every part of every tooth, they have arrived at a point where good has ceased and harm commences. It is the commonest of things in one's experience to see the roots uncovered by this vicious scrubbing process, particularly those of the exposed cuspids, and the practice being continued on the soft dentine, producing concavities that soon become cavities, and finally unsightly fillings. It is bad enough when we must have this state of things to endure as the result of specific erosion,—the *bête noire* of every conscientious operator. It is, I am persuaded, unjustifiable to allow patients to deliberately produce it mechanically. If my patients have any extra energy to dispose of, I beg of them to give it to flossing the approximal surfaces, and often say to them that if they slight one of the two things, the floss or the brush, slight the brush by all means, as the surfaces touched by the brush are all more or less taken care of by the forces constantly at work in the mouth,—*e.g.*, the action of mastication, of the lips, cheeks, and tongue, aided by the washing effect of the flowing saliva, etc.,—but that the most dangerous places, the approximal spaces, which are needing the most of artificial care, not only receive none from the brush, but are actually further burdened by the brush sweeping more material into them.

*Dr. Elliott.*—There seems to be a good deal of difference of opinion among us, but if the matter be looked into closely, it will be found that we are all of one mind. I did not say that the brush

35

was not capable of doing injury if injudiciously used. I have seen injury that may have come from the brush, but that is an injudicious use of a good thing. If properly used, it is impossible to do damage with it. We often see the gum pushed away from the teeth, but that is not as dangerous as the loosening of the teeth would be, because, even if there is a recession, the tooth can still be retained in good condition. So it is really a difference in the *modus operandi*. The point that to me is important is that the gums do not get enough work to do, and we should make up for that by stimulating them with a stiff brush.

*Dr. Hodson.*—I do not think I ever saw inflamed gums—barring, of course, those due to constitutional causes—that were not so as a a legitimate consequence of underlying tartar. Upon a thorough removal of this cause—an operation, by the way, which is seldom given the exhaustive attention which it merits—the gums tend at once, and spontaneously, to a healthful condition. I do not recognize any inherently diseased condition of the gums from non-work *per se*. As well might we say such a thing of the mucous membrane lining the maxillary sinus, or the nares, or anywhere in the whole tract where it is spread over a bony foundation, and so in corresponding circumstances with that in the mouth.

*Dr. McNaughton.*—Is tartar not always preceded by a little inflammation of the gums?

*Dr. Elliott.*—Feed a dog on soft food for three months, and examine his gums, and then put him on hard food, give him bones, etc., and in another month you will find an improved condition.

*Dr. Hodson.*—It is a case of restored health.

*Dr. Elliott.*—Restored health from use.

*The Chairman.*—We will now hear from Dr. W. St. George Elliott, the chairman of the Committee on Current Literature.

### REPORT ON CURRENT LITERATURE.

*Dr. Elliott.*—Certainly the harmonious relationship of the human features is not only worthy of the deepest study, but should receive that recognition from the entire profession to which it is entitled. There are, no doubt, fixed laws which control the size and shape of the teeth, their relationship to the features and temperament. Unfortunately, these laws are but little understood by us, and so a contribution on the subject is always welcome. Dr. Norman Broomell has given us in the January *Dental Cosmos* a very interesting paper on this subject most beautifully illustrated.

Dr. Broomell, unfortunately, does not in this paper give any practical points to govern us in our work. This, no doubt, he may be able to do at some future time. The main object of the paper is to make us familiar with the four great types of temperament, the bilious, the lymphatic, sanguinary, and nervous, and having fully recognized them in our patients, to make such use of the knowledge in artificial work as will lead to a satisfactory and artistic result. It may not be an exaggeration to say that ninety per cent. of the prosthetic work we meet with shows either gross ignorance or a flagrant disregard of the harmonious laws of nature. I would say just here that I believe, as a rule, the profession are not to blame for this; the fault lies with our patients. I remember some twenty-five years ago being consulted by an elderly lady who wished to have a partial upper denture made. The few remaining natural teeth were sufficient guide, I thought, to enable me to construct a plate that would have teeth in harmony with her features. On completing the case I thought myself entitled to some commendation for the results; but, on giving the patient the mirror, what was my surprise when she said, "Oh, those teeth will not do; they are like horses' teeth; my teeth were small and white;" although I had the evidences to the contrary before me. Some patients are honest enough to tell us that when they are getting new teeth they might as well have nice ones.

Dr. Barrett, of Buffalo, read an excellent paper on a similar subject before the American Dental Association, at Saratoga, last summer, treating the subject from an anatomical point of view, showing the necessity of allowing proper play to the oral muscles, and so making the denture that it will not interfere with the muscles of expression.

Dr. Weld's paper before the Odontological Society appears in the same number of the *Dental Cosmos*, and we have had some chemical demonstrations at the Dwinelle. Briefly, the chemico-metallic method consists of filling root-canals with a broach made of silver, tin, and zinc. This is oxidized by the application of aqua regia or its equivalent. This oxidation, together with the generation of gas and the change in the canal contents resulting from the chemical action, sterilizes the root, while the alloyed broach acts as a filling. In 1875, and subsequently, I used fine copper wire, without acids, for this purpose with fairly satisfactory results, but soon abandoned it for the same reasons I would be inclined to criticise Dr. Weld's method,—*i.e.*, the practical impossibility of so gauging the length of the wire as to just fill the canal and no more, although I recognize the possible value of the chemical combination.

Dr. Farrar has a short article in the January number of the *Dental Cosmos* on some points in the specialty he has done so much for.

It is his belief that in the correction of protruding upper teeth the teeth swing upon their middle part, the fulcrum being a short distance above the necks of the teeth, and sometimes the roots in moving forward move the upright plates of the maxillary bone. This is a very interesting point, and I hope Dr. Farrar will give us some proof of it. I certainly think he is mistaken. He also states that "the generally accepted impression that considerable extra space in regulating can be gained by widening the dental arch is erroneous." I think he is mistaken. It is a common practice in London for dentists to use the Coffin expansion plate in the first stage of regulating to gain the required space; indeed, I have known them to correct slight irregularities in this way alone, the teeth correcting themselves when liberated from lateral pressure.

Naturally, Dr. Farrar with his experience believes in the advantages of extraction to facilitate regulation. I remember Dr. Norman W. Kingsley showing me a case many years ago that he had successfully regulated, taking some two years, and stating that he subsequently found that he could have accomplished the same thing in quarter of the time had he had recourse to extraction at an early stage.

Dr. Register, of Philadelphia, read a paper before the Pennsylvania State Dental Society, in which he takes up the investigations of Drs. Black and Miller as to the cause of caries, and gives us some valuable tables of the comparative durability of his own operations with gold, alloy, and phosphate. Dr. Register deserves much credit for his honesty in giving us this table, for most of us would not like to tell our patients that our gold fillings only averaged 7.73 years and amalgam a little over five. I fancy it is nevertheless true, if, indeed, they last so long.

Dr. Black in his investigation of the comparative hardness of different teeth, if I remember aright, distinctly states that by hardness he does not mean resistance to cutting-instruments. One can readily conceive a tooth to be strong under compression, rich in inorganic matter, and yet its molecules so loosely held together that they do not resist cutting-instruments well. Terra-cotta and porcelain may stand compression well, but there is an immense difference if they are cut into. Consequently I infer that when micro-organisms are once located on a tooth, the rapidity of decalcification by acid secreted by the organism will be found to be in direct proportion to the hardness, as judged by cutting-instruments and not by the proportion of lime-salts.

In *Welsh's Monthly* there is an article by Fletcher, of Warrington, England, a criticism on Dr. Black's article on "Amalgams." It seems that Dr. Black in one of his analyses gives the amount of mercury squeezed out to the second decimal. Fletcher contends that the amalgam is of no value, as the mercury was not mercury, but an alloy containing an excess of that metal, which is doubtless true. He states that an excess of mercury having been added, the squeezing out of the so-called mercury made the results so erratic that it was entirely discarded by him at an early stage. No more mercury should be put into an alloy than is necessary for proper working, which can be either wet or dry.

In the March number of the *Dental Digest* Dr. Black replies to the editorial comment on his article on the proper shaping of approximal fillings. It will be remembered that in Dr. Black's paper he advised the cutting away of the entire approximal surface down to the gum, so that the margin of the finished filling will be as far removed from the centre of the approximate space as possible, for the purpose of making it self-cleansing by the motion of the tongue and the food. Dr. Crouse in his criticism had stated that the preparation of the cavity and filling and finishing when conscientiously performed must consume from four to six hours of time, independent of that taken in securing the necessary space, besides requiring an amount of skill and good judgment not possessed by the majority of operators.

Dr. Black in his reply reiterates his statement, and believes that when dentists possess a clear apprehension of the principles involved, bold cutting upon definite lines is something quite different from feeling one's way to possible sound margins, and is done in much less time and with less worry to the patient. As to the cost of such operations, Dr. Black says the best is the cheapest in the end, costing less than several less efficient measures.

In the March number of the *Dental Cosmos* we have one of the most interesting and important papers that has been published in a long time. It is the paper by Dr. Leon Williams, of London, on "The Pathology of Enamel." I had the pleasure of hearing the paper read and seeing the lantern illustrations. No paper written on this subject seems to have advanced our knowledge of caries of the teeth to the same extent. Briefly stated, Dr. Williams proves by his illustrations that caries is the result of acid generated by a patch of micro-organisms lying on some protected surface of the enamel, the organisms themselves following in the line of decalcification. He also shows that the chemical or physical formation of the tooth has but little to do with the frequency of decay; that defective tooth-structure *per se* is not necessarily the point of attack by these organisms; that the teeth of animals, as shown by the slides, are often defective in structure and yet do not decay.

Dr. Bryant, of Washington, describes an excellent combination, consisting of a gold plate solidly attached to the natural teeth and yet removable; upon this gold plate a continuous gum plate is attached by means of a bolt and nut and gutta-percha. The whole is removable by the patient, and in case of fracture the two parts can be readily detached.

In the March number of the INTERNATIONAL DENTAL JOURNAL the editor, Dr. Truman, has something to say in regard to serrations on our pluggers. From personal experience I think he is right in his views, that at first we had very shallow serrations, then coarse, and now again shallow, and I think some of our young men should make some experiments to determine the scientific facts. My own belief is that sharp serrations should be used on the body of the filling, not necessarily coarse but sharp, and that near and on the surface fine, shallow serrations only should be used. I have seen many fillings destroyed by a lack of cohesion at some point, which would not have occurred had suitable serrations been used. I do not believe it is the serrations that produce pitting, but lack of condensation.

A gentleman came to me some weeks ago for a simple biproximal filling. That filling was knocked out twice within three weeks, and in each instance pieces of the gold had detached themselves, one from the other, until finally I used very coarse serrations, and then I found that I was not relying on a property of which I was ignorant,—that is, the cohesion of the gold.

In the *British Journal of Dental Science* there is a short article by Dr. Rosenthal, I suppose of Liége, Belgium, on the treatment of pyorrhœa by silver bands. He found out accidentally that the bands exerted a most beneficial influence, doubtless on the theory that the metallic salts, the result of chemical action on the silver bands, act as germicides, and being always present the effect is permanent. A theory worth trying.

Dr. Rosenthal, many years ago, introduced a cure for alveolar abscess. He brought it over to London, and finding that I had a suitable ejector, that gave a vacuum of about twenty-one inches, he came to me, and we gave a demonstration at my office. It consisted of an ejector for getting the exhaust and a syringe with a three-way cock. With the ejector in the tooth and a bit of wax to make it air-tight, he would turn the faucet and exhaust all the air. A medicament would then be thrown in, the faucet turned again to exhaust that, and so on as many times as desired. He presented me with the apparatus, but I did not find it practicable.

DISCUSSION.

*Dr. Lord.*—I think we may get a great deal of interest and instruction of one kind and another from the reports of the Committee on Recent Literature, calling our attention to certain articles and giving some suggestions in regard to them. Perhaps if we had read the articles we would give them a second reading, and if we had not read them, we would be likely to look them up.

I have read Dr. Black's paper alluded to in the report, and I certainly felt a great deal of regret and disappointment that a man of his intelligence should advance such views; they seemed to me simply absurd.

I was greatly interested in Dr. Crouse's criticism of the article in question. It did only justice, and is calculated to be very useful. The terms which I use may seem a little harsh, but such views coming from a man of Dr. Black's intelligence seem dreadful and are likely to do much harm.

The chairman of the committee, in reporting on prosthetic dentistry, said that sometimes he thought failures were not due to the dentist so much as to the patient. It has always been my feeling that dentists should not consult their patients, at least not to much extent, in regard to the detail of artificial dentures. The dentist ought to know a great deal better than the patient in regard to the color, expression, and everything connected with the fitting or adaptation for the greatest usefulness; we cannot trust our patients to decide those questions.

*Dr. Brockway.*—I am glad to see this practice introduced, of having a summary from the dental journals prepared for us at the meetings. I have long been of the opinion that it was a source of information and instruction that societies were very remiss in not improving. It is almost impossible for us to read all the journals; many of us are not able to afford them all, and often we have not the time to read them; but if we can have a review and selection from the various journals presented at our meetings, it will be a source of very great advantage.

*The Chairman.*—We will now listen to the paper of Dr. L. P.

Bethel, of Kent, Ohio. The subject is "The Use of Silver Salts in the Treatment of Root-Canals." The photomicrographs of the specimens sent by Dr. Bethel were kindly made for us by Dr. Andrews, of Cambridge, and they will be thrown upon the screen in illustration of the points of the paper.

The Secretary read Dr. Bethel's paper.

(For Dr. Bethel's paper, see page 485.)

DISCUSSION.

*The Chairman.*—Gentlemen, Dr. Bethel's paper is now before you for discussion.

*Dr. E. A. Bogue.*—The subject for consideration this evening must interest us all, and as I read the discussions on this same subject in our recent literature, there are a few points upon which clear pronouncements have not been made.

When we speak of treating a root-canal, we are not apt to carefully specify that this is a canal in which a pulp spontaneously died and lay dead for from one to five years, without a suspicion on the part of its possessor as to its real condition, until an abcess had begun to form at one or two of the apices of the roots, accompanied by extensive pericemental inflammation. Similar inaccuracy may be expected if the pulp died from the effects of an oxychloride of zinc capping, and was found, to the surprise of the operator, atrophied, when he, by chance, opened into the pulp-chamber.

A third condition that might be in the mind of one who described the process would be that of a pulp-canal from which the pulp had been extracted piecemeal, so far as it could be reached, and the rest (in blissful ignorance on the part of the operator, generally not so blissful on that of the patient) left, because it could not be reached or because it was supposed it could do no harm.

A fourth description of a pulp-canal would be one whose pulp had been destroyed by the application of an escharotic, like arsenic, for example, which had been allowed quietly to rest for ten or twelve days, until the slough between the dead and living portions had taken place, and then, before disintegration had ensued, the pulp had been entirely removed.

Now, as I conceive, these four conditions are quite different and would require different treatment.

The questions arising about the use of coagulants would naturally present themselves, if one were contemplating the treatment and filling of the two conditions last described,—because if albuminoids in any shape are to be met with and taken account of in our treatment of root-canals, one would expect them to be found either where the pulp had been wholly removed through the operation of an escharotic and its consequent slough, or when the pulp had been picked out piecemeal as far as our instruments can reach and the remainder, with the processes leading into the dentinal tubes, left.

In the case of the second root described, where atrophy of the pulp had taken placc, we have the organic contents of the dental canal and dentinal tubes present in as nearly a mummified condition as may be.

While in the first-described roots we have a mass of putrefactive decomposition, probably saturating the dentinal tubes from a quarter to a half their length, and we have the denuded apex of the root from which the pericemental covering has been stripped by the processes of a forming abscess.

In the *Transactions of the American Dental Association* for 1896, Dr. L. P. Bethel presented a paper on lining root-canals with nitrate of silver by cataphoresis that has interested me much, and the discussion upon that paper is equally interesting.

Dr. Bethel, I judge, means to start from a point at which all soreness or tenderness of the teeth or roots, to pressure, has ceased. He also leads us to suppose that he has washed out the canals and rendered them as clean as washing can make them. Then he carefully states that the object of his experiments is to find a means of treating root-canals too small, or tortuous, or flat to admit a broach, and where it is doubtful about inserting a protecting root-filling.

Dr. Bethel leads us to infer that our ordinary root-fillings that we put into the six front teeth, some few bicuspid roots, and the palatal roots of the upper molars, and others that are accessible, are efficient, and he modestly presents a method that was entirely new to me of treating and filling or lining the inaccessible roots, for it seems from appearances that this cataphoric driving of nitrate of silver into a tortuous root containing certain organic matters results in a decomposition of the surplus of the silver salt into metallic silver on the one side and an insoluble substance on the other, the product of the chemical union of the salt with the organic matter, and that these very small roots are filled. I say this about the metallic silver from what I have read and been informed, for I have not seen the specimens. But one has only to read the abstract of Dr. Credé's paper in the February number of the INTERNATIONAL DENTAL JOURNAL to see the action of the in fected products of bacterial life upon silver; if, then, lactate of silver is so easily generated as is shown in that paper, we see that we have in Dr. Bethel's method not only a mechanical filling which seems the best adapted to prevent the entrance of septic matter, but that if there is a minute mechanical plug of metallic silver, it would in the presence of an infected condition generate lactate of silver, the powerful antiseptic that in a watery solution of 1 to 4000 destroys all bacteria within ten minutes.

Dr. Bethel in his paper thus calls attention to a fifth classification of roots needing to be treated and filled,—viz., roots too small, thin, or tortuous to be reached by ordinary methods. He then proceeds to give us a method of both treating and filling these minute roots. But he *nowhere* recommends silver nitrate in solution as a good thing with which to treat or line any of the six or eight front teeth. Nor does he say that because silver nitrate will not discolor teeth save superficially when applied as a caustic externally, where the branchings of the dentinal tubuli are smallest, and where the currents so far as we know are centrifugal, therefore it must be superficial in its action when applied in the pulp-chamber in solution at the trunk ends of the dentine tubes, and with the electric current behind it.

*Dr. Elliott.*—Some months ago I procured some of the lactate of silver and used it in half a dozen cases. With me the cases were all unfortunate.

*Dr. Davenport.*—I move a vote of thanks to Dr. Bethel for his paper and for the slides which he prepared for its illustration. Also allow me to include Dr. Andrews, of Cambridge, who has so kindly photographed the slides for use this evening.

Carried unanimously.

Adjourned.

S. E. Davenport, D.D.S., M.D.S.,
*Editor The New York Institute of Stomatology.*

